# Cancer-Therapy-Induced Cardiotoxicity: Results of the Analysis of the UK Yellow Card Adverse Drug Reaction (ADR) Reporting

**DOI:** 10.3390/cancers16244223

**Published:** 2024-12-18

**Authors:** Stefanie Ho Yi Chan, Deborah Layton, Sherael Webley, Sam Salek

**Affiliations:** 1 School of Life and Medical Sciences, University of Hertfordshire, Hatfield AL10 9AB, UK; 2 Department of Pharmaceutics, UCL School of Pharmacy, London WC1N 1AX, UK; 3 PEPI Consultancy Limited, Southampton SO53 1GR, UK

**Keywords:** non-small cell lung cancer, non-small cell lung cancer treatments, cardiotoxicity, cardiovascular adverse events, pharmacovigilance, disproportionality analysis

## Abstract

**Background:** Non-small cell lung cancer (NSCLC) is a predominant cause of oncological mortality in the United Kingdom. There is a diverse spectrum of therapeutic options available, such as chemotherapies, targeted therapies and immunotherapies, which have significantly advanced patient prognoses. However, despite these advancements, there is an escalating concern regarding the potential cardiotoxic effects associated with these treatments. **Objectives:** This study aimed to explore the association between non-small cell lung cancer treatments and cardiotoxicity. **Methods:** A pharmacovigilance study was conducted utilising the UK Yellow Card System. The proportional reporting ratio (PRR) and reporting odds ratio (ROR) were calculated to detect signals. **Results:** Among the 56 shortlisted NSCLC drugs, the total number of adverse events reported was 128,214 with 6133 reports being cardiovascular adverse reactions. Among all the drugs analysed, alectinib demonstrated the highest ROR and PRR values, indicating the strongest signal for potential cardiovascular adverse events. **Conclusions:** This result was comparable to previous studies which also detected a signal of alectinib related to cardiovascular events using the WHO pharmacovigilance database, VigiBase. However, clinical studies demonstrated that alectinib largely improved progression-free survival (PFS) and overall survival in patients. Therefore, it is important to continue monitoring the real-world use of alectinib, so that a benefit–risk balance can be maintained.

## Simple Summary

Non-small cell lung cancer (NSCLC) is a leading cause of cancer-related deaths in the United Kingdom. Advances in treatments such as chemotherapy, targeted therapies, and immunotherapies have significantly improved outcomes for patients. However, these treatments have raised concerns about their potential heart-related side effects. This study investigated this issue using the UK’s Yellow Card System, which collects data on drug side effects. The study examined adverse event reports for 56 NSCLC drugs, finding 128,214 adverse events, of which 6133 were cardiovascular issues. Alectinib emerged as the drug with the highest reported risk of cardiovascular problems, aligning with findings from other databases like the WHO’s VigiBase. Although alectinib has been shown to significantly improve the survival rate of cancer patients, continuous monitoring is essential to ensure a benefit–risk balance can be maintained.

## 1. Introduction

Lung cancer remains a major health challenge in the United Kingdom, as evidenced by its incidence and mortality rates. In 2022, the GLOBOCAN database, released by the International Agency for Research on Cancer (IARC), reported 454,954 new cancer cases and 181,807 cancer deaths in the UK. Specifically, the age-standardised incidence and mortality rate were 307.8 and 98.3, respectively, with 1,435,322 five-year prevalent cases [1]. Lung cancer accounted for 50,700 new cases in the UK in 2022 alone, accounting for 11.1% of all cancer diagnoses, making it the third most common type of cancer after breast and prostate cancer. It was also the leading cause of cancer mortality, with 35,394 deaths, representing 19.5% of all cancer deaths. The cumulative risk for an individual to be diagnosed with lung cancer and dying from lung cancer in the UK were 3.7% and 2.3%, respectively. The five-year prevalence of lung cancer was 60,603 cases, with a proportion of 4.2 cases per 100,000 individuals [1].

Non-small cell lung cancer (NSCLC) is the most common type of lung cancer, and it consists of several subtypes, each with unique characteristics, etiologies, and treatment responses. Adenocarcinoma, squamous cell carcinoma, and large cell carcinoma are the most prevalent among all the subtypes. During the past three years, PD-1/PD-L1 inhibitors and kinase inhibitors have been included as the standard care for NSCLC treatments [2]. Between 2017 and 2020, spending on NSCLC treatments increased by 50% to USD $16.6 billion, with three-quarters of this growth attributed to PD-1 inhibitors [3]. On the other hand, there are notable advancements of NSCLC targeted therapies in the development and utilisation of EGFR and ALK inhibitors, which are majorly 2nd- and 3rd-generation small-molecule therapeutics, aimed at enhancing clinical outcomes through improved safety profiles and increased effectiveness against common resistance mutations [3]. Moreover, the COVID-19 pandemic heightened interest in mRNA vaccines. Decades of prior research in mRNA vaccines in oncology contributed to the rapid development of COVID-19 vaccines. In 2022, there were 21 oncology mRNA vaccines in development with 4.8% intended for NSCLC [2].

While these treatments have significantly improved outcomes for NSCLC patients, there is growing concern regarding their potential impact on cardiovascular events. Cardiotoxicity refers to the detrimental effects of cancer treatments on the heart and blood vessels, which can lead to various cardiovascular complications. While the primary goal of cancer treatment is to eliminate or control the malignant cells, the unintended consequence of some therapies is damage to the heart, hence compromising its ability to function optimally. Cardiotoxicity was initially documented in 1967 during the administration of daunomycin, an anthracycline, in leukaemia patients [4]. Subsequent to this, there was an escalation in reports of anthracycline-induced cardiotoxicity in the early 1970s. Following this period, an increasing trend in reported cases of cardiotoxicity associated with various oncological agents has been observed. The incidence of cardiotoxicity varies across different treatment modalities, and its impact can range from mild, reversible effects to severe and irreversible damage [5,6].

With growing concerns about the cardiotoxic effects of cancer treatments, pharmacovigilance systems play a vital role by detecting, evaluating, and mitigating these adverse effects, making pharmacovigilance essential for ensuring patient safety. Pharmacovigilance, the practice of monitoring the safety of medicines, is crucial for identifying adverse effects not detected during pre-marketing studies [7]. Regulatory agencies use real-world data (RWD) to quickly identify potential safety issues, which can significantly reduce the health risks associated with new drugs and devices. It provides evidence needed for modifying drug labels, updating safety guidelines, and informing public health policies [8].

By contributing to the monitoring and evaluation of the safety profiles of medicines and vaccines, the UK Yellow Card System is an integral part of the country’s pharmacovigilance framework. The scheme, managed by Medicines and Healthcare products Regulatory Agency (MHRA), operates on a voluntary reporting basis. The Yellow Card scheme allows healthcare professionals, patients, and caregivers to report suspected adverse reactions voluntarily. Reports can be submitted online, via a paper form, or through a dedicated mobile application. The system collects information on the suspected drug, the reaction, patient demographics, and other relevant details. Reports can be submitted anonymously, ensuring confidentiality and encouraging reporting without fear of reprisal. The ease of reporting empowers healthcare professionals and patients to actively participate in drug safety monitoring, contributing to a more comprehensive understanding of medication risks [9].

This study aimed to explore the association between non-small cell lung cancer treatments and cardiotoxicity by conducting disproportionality analyses which are used to detect safety signals. This objective aligns with ongoing efforts to improve cancer care by integrating real-world evidence into the understanding of treatment-related risks. By providing insights into the real-world occurrence of cardiotoxicity, the findings from this analysis will contribute to enhancing clinical awareness, guiding risk management strategies, and ultimately improving cancer patient outcomes.

## 2. Methods

This study was a disproportionality analysis with secondary use of the Yellow Card database—a spontaneous reporting system—in the United Kingdom. All lung cancer (ICD-10 code: C34) drugs (*n* = 56) available within the Yellow Card database were shortlisted (Table 1). Etoposide, ifosfamide, lurbinectedin and topotecan are approved for treating small cell lung cancer (SCLC); however, they are also used to treat NSCLC off-label, so they were also included.

The data downloaded were individual drug analysis profiles (DAPs). The medicines were listed by the name of the active ingredient, and not by brand name. Each DAP consisted of a list of all suspected adverse drug reactions (ADRs) that had been reported by healthcare professionals, patients and pharmaceutical companies to the MHRA via the Yellow Card scheme. This was carried out for all licensed medicines as part of routine PV. There was a time lag ~1 month from receipt of a report to it being included in the DAP. The most recent available data at the time of data extraction (October 2023) were up until August 2023. Medical Dictionary for Regulatory Activities (MedDRA) version 26.0, March 2023 was used (Available from: https://www.meddra.org/ (accessed on 28 October 2023)). After data extraction, data were mapped using Microsoft Access/Microsoft SQL Server. Data analyses were then carried out using R (version 4.3.3). A list of variables extracted from the Yellow Card database is listed in Table S1.

The counts and types of cardiotoxic events in all patients within each cancer drug were summarised. Descriptive analyses were conducted using number and percent within each category with 95% confidence intervals (whenever appropriate) for categorical variables, and mean (standard deviation [SD]), median (Q1, Q3), and minimum and maximum for continuous variables. Sub-group analyses stratified by age and sex were also conducted where possible.

Disproportionality analyses in spontaneous monitoring, i.e., proportional reporting ratio (PRR) and reporting odds ratio (ROR), were used to detect signals of adverse cardiovascular effects of the shortlisted NSCLC drugs. It involves statistical methods that compare the observed frequency of reports of a specific drug–event pair to the frequency expected if there is no association between the drug–event pair [7,10]. Cardiovascular events of interest were defined as cardiac disorders or vascular disorders (code: ‘card’ or ‘vasc’) under System Organ Class (code: SOC_ABBREV).

The proportional reporting ratio (PRR) measures the proportion of all adverse event reports for a given drug that are for a specific adverse event and compares this to the proportion for all other drugs. The PRR gives an indication of whether the observed proportion is significantly higher than expected. A PRR value ≥ 2, a chi-squared (*x*^2^) value ≥ 4, and at least three reported cases suggest a signal [11]. The PRR was calculated as follows:

PRR = (a/(a + b))/(c/(c + d))(1)

where a was the number of reports of a specific adverse event for the drug of interest, b was the number of reports of all other adverse events for the drug of interest, c was the number of reports of the specific adverse event for all other drugs, and d was the number of reports of all other adverse events for all other drugs.

The reporting odds ratio (ROR) compares the odds (not the proportion) of reporting a specific adverse event for the drug of interest to the odds of reporting that event for all other drugs. ROR can provide an estimate of the relative risk, and it can be accompanied by a confidence interval to assess the precision of the estimate. When the lower limit of the 95% confidence interval (CI) is larger than 1, then ROR is considered significant [12]. The ROR is calculated as follows:

ROR = (a/b)/(c/d)(2)

where a, b, c and d were the same as for the PRR.

## 3. Results

The total number of adverse events reported for the shortlisted 56 drugs was 128,214. Among those, 6133 reports were adverse cardiovascular reactions. Table 2 shows an overview of the total number of adverse event reports received for each drug, and the number of cardiovascular events reported for each corresponding drug. Out of the 56 shortlisted drugs, 6 drugs (amifostine, lurbinectedin, pralsetinib, ramucirumab, sotorasib, and veliparib) had no reported cardiovascular adverse events. In addition, alectinib and epirubicin were the only two drugs which received over 10% of cardiovascular adverse event reports.

Table S2 shows the patients’ demographics, including age and gender, and seriousness of each cardiovascular adverse event reported by drug. Seriousness was divided into non-serious, serious and fatal where fatal was a subset of serious.

**Figure 1:**
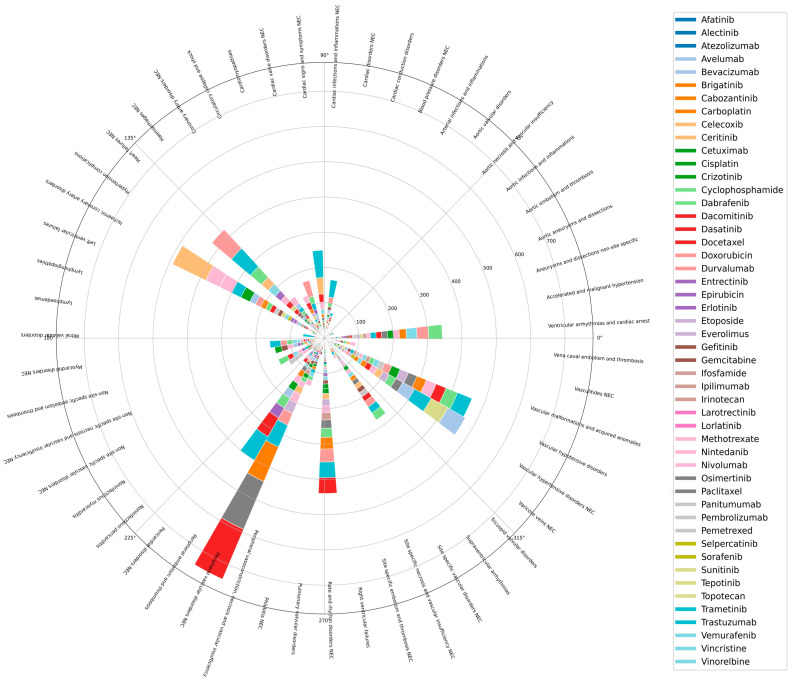
An overview of MedDRA High-Level Term (HLT) reported under cardiology or vascular class across the 50 shortlisted drugs for which cardiovascular adverse drug reaction reports were available. (NEC refers to ‘Not Elsewhere Classified’).

This circular barplot presents an overview of MedDRA High-Level Term (HLT) re-ported under cardiology or vascular class (Figure 1). The top 5 HLT adverse events are peripheral vascular disorder, ischaemia coronary artery disorder, vascular hypertensive disorder, vascular hypotensive disorder and rate and rhythm disorder.

**Figure 2:**
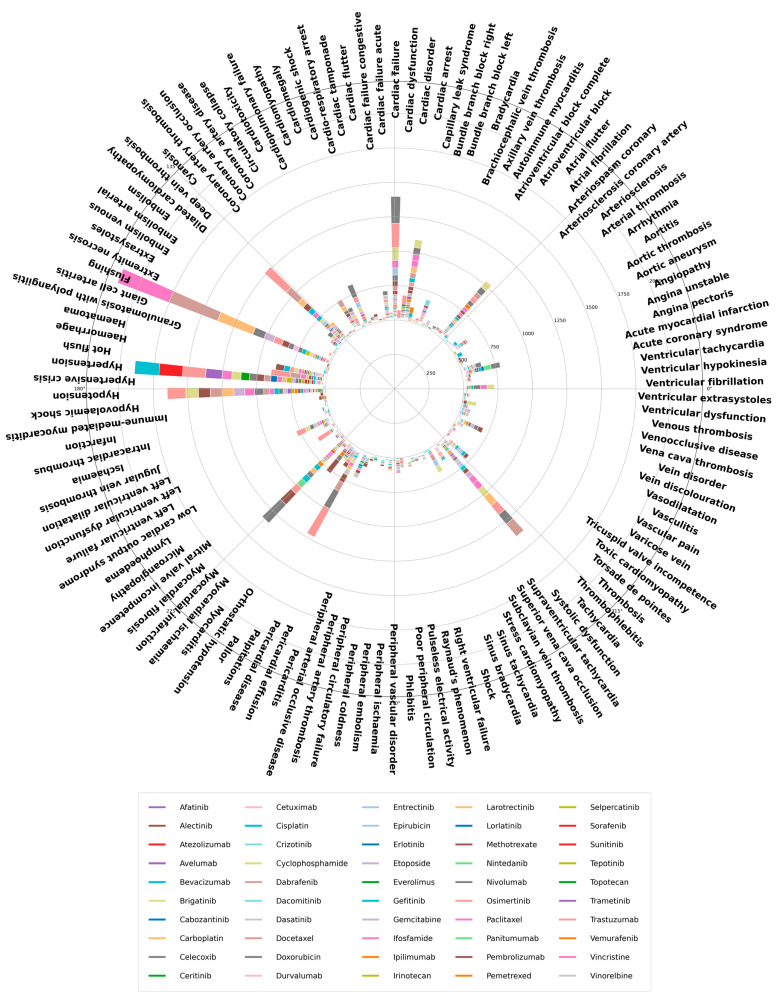
An overview of MedDRA Preferred Term (PT) reported under cardiology or vascular class across the 50 shortlisted drugs for which cardiovascular adverse drug reactions reports were available.

The following plot utilises Preferred Terms (PTs) to offer more detailed and specific insights into the individual adverse events, thereby enhancing the granularity and specificity of the safety signal detection. This circular barplot presents an overview of MedDRA Preferred Terms (PTs) reported under cardiology or vascular class (Figure 2). This only included reactions with a minimum of 5 cases across the 50 shortlisted drugs with suspected cardiovascular adverse events.

The top 10 most frequently reported cardiovascular reactions (Preferred Terms Level) were flushing (*n* = 541), followed by hypertension (*n* = 455), hypotension (*n* = 376), cardiac failure (*n* = 294), tachycardia (*n* = 291), myocardial infarction (*n* = 273), deep vein thrombosis (*n* = 250), palpitations (*n* = 237), cardiac arrest (*n* = 195) and atrial fibrillation (*n* = 173). Paclitaxel received the highest number of counts (*n* = 131) in flushing. In addition, reports of flushing were received for 28 other drugs. The highest count of hypertension was observed in bevacizumab (*n* = 58), and 35 other drugs also received reports of hypertension. Trastuzumab had the highest count of adverse reactions in hypotension (*n* = 44), deep vein thrombosis (*n* = 69), and palpitations (*n* = 77).

**Figure 3:**
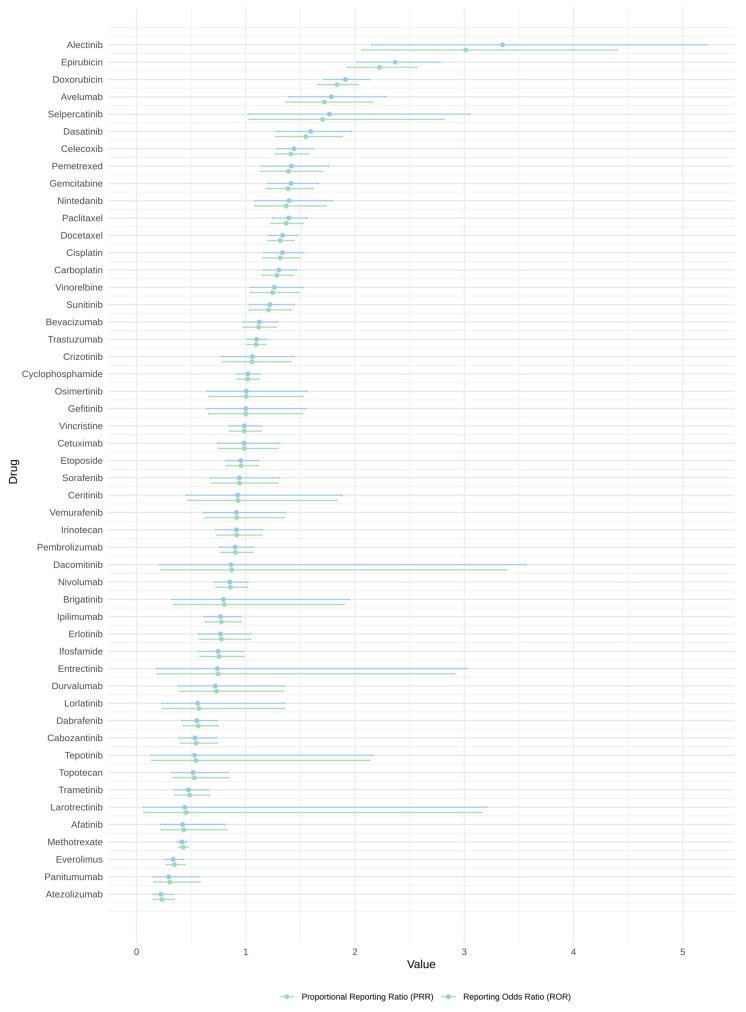
A forest plot showing both ROR and PRR values with the corresponding 95% CI of each drug.

The reporting odds ratio (ROR) provides insights into the odds of cardiovascular adverse events for each drug. The forest plot (Figure 3) shows the ROR values of each drug and its corresponding 95% CI. Seventeen drugs (i.e., alectinib, avelumab, carboplatin, celecoxib, cisplatin, dasatinib, docetaxel, doxorubicin, epirubicin, gemcitabine, nintedanib, paclitaxel, pemetrexed, selpercatinib, sunitinib, trastuzumab and vinorelbine) had an ROR value (lower limit of the 95% CI) of ≥1, which indicated a signal. Alectinib had the highest ROR value of 3.35, indicating a higher odds of cardiovascular adverse event reports compared to other drugs. The confidence interval ranged from about 2.15 to 5.21, suggesting a significant association, although there was some variability in the estimate. Atezolizumab had the lowest ROR value of 0.22, suggesting fewer cardiovascular adverse event reports than expected. Among the 17 drugs which detected a signal with cardiovascular adverse events, traditional chemotherapies included carboplatin, cisplatin, docetaxel, doxorubicin, epirubicin, gemcitabine, paclitaxel, pemetrexed, vinorelbine; targeted therapies included alectinib, dasatinib, nintedanib, selpercatinib, sunitinib; and immunotherapies included avelumab and trastuzumab. Celecoxib also detected a signal; it is often used in combination with chemotherapies.

The proportional reporting ratio (PRR) provides a different perspective on the same issue, focusing on the proportion of adverse event reports for each drug. The forest plot (Figure 3) shows the PRR values and corresponding 95% CI of each drug. Alectinib also had the highest PRR value, reinforcing the signal detected by the ROR analysis. The 95% confidence interval ranged from 2.06 to 4.40, which supported the presence of a strong signal for cardiovascular adverse events. However, according to the criteria of signal detected by PRR, cardiovascular adverse event signals were detected in alectinib (PRR = 3.01, *x*^2^ = 32.36, No. of cases = 23) and epirubicin (PRR = 2.22, *x*^2^ = 118.91, No. of cases = 173) only, as opposed to the 17 drugs detected by ROR analysis. Atezolizumab again showed the lowest PRR value of 0.23, consistent with the ROR findings.

Among all the drugs analysed, alectinib demonstrated the highest ROR and PRR values, indicating the strongest signal for potential cardiovascular adverse events. This suggested that reports of cardiovascular events associated with alectinib were more frequent than expected when compared to other drugs. It is important to note that while these metrics can signal potential safety issues, they do not establish causality. Further investigation is required to determine whether the associations are due to the drugs themselves, other confounding factors, or a combination of both.

The sub-group analysis of this study included stratification by 10-year age bands and by sex. A total of 16 (carboplatin, cisplatin, cyclophosphamide, docetaxel, doxorubicin, epirubicin, etoposide, gemcitabine, ifosfamide, irinotecan, methotrexate, paclitaxel, pemetrexed, topotecan, vincristine, vinorelbine) of the 56 drugs were chemotherapy drugs. Signals detected include males of age 30–39 in docetaxel (ROR = 9.96, 95% CI = 1.82–54.37; PRR = 6.97, *x*^2^ = 10.74), males of age 80–89 in doxorubicin (ROR = 13.28, 95% CI = 4.73–37.33; PRR = 8.37, *x*^2^ = 40.85), males of age 80–89 vincristine (ROR = 6.64, 95% CI = 2.64–16.74; PRR = 5.23, *x*^2^ = 21.54) and males of age 80–89 in vinorelbine (ROR = 5.97, 95% CI = 1.64–21.71; PRR = 4.83, *x*^2^ = 9.55). Moreover, signals were only detected in females (with the exception of males aged 0–9) for topoecan.

Twenty-seven (afatinib, alectinib, bevacizumab, brigatinib, cabozantinib, ceritinib, cetuximab, crizotinib, dabrafenib, dacomitinib, dasatinib, entrectinib, erlotinib, everolimus, gefitinib, larotrectinib, lorlatinib, nintedanib, osimertinib, panitumumab, selpercatinib, sorafenib, sunitinib, tepotinib, trametinib, trastuzumab, vemurafenib) of the shortlisted drugs which were targeted therapies reported cardiovascular adverse events. Signals were detected in males aged 50–59 in alectinib (ROR = 13.95, 95% CI = 5.31–36.66; PRR = 8.62, *x*^2^ = 49.44), males aged 70–79 in gefitinib (ROR = 5.24, 95% CI = 1.96–14.04; PRR = 4.36, *x*^2^ = 13.58), females aged 60–69 (ROR = 4.10, 95% CI = 1.82–9.26; PRR = 3.57, *x*^2^ = 13.60) as well as males aged 70–79 in osimertinib (ROR = 3.62, 95% CI = 1.25–10.51; PRR = 3.22, *x*^2^ = 6.42), males aged 60–69 in selperacatinib (ROR = 8.53, 95% CI = 2.21–33.01; PRR = 6.27, *x*^2^ = 13.96), females aged 80–89 in sunitinib (ROR = 3.46, 95% CI = 1.20–10.02; PRR = 3.10, *x*^2^ = 5.97) and females aged 20–29 in vemurafenib (PRR = 20.91, *x*^2^ = 19.91). It was also detected that certinib, dacomitinib, entrecitinib, larotrectinib, tepotinib might be related to females only while lorlatinib might only be related to males.

Six (atezolizumab, avelumab, durvalumab, ipilimumab, nivolumab and pembrolizumab) of the shortlisted treatments were immunotherapies. There were no particular trends or patterns observed as a therapeutic class overall. However, signal was detected in females aged 90–99 in pembrolizumab (ROR = 19.91, 95% CI = 2.80–141.38; PRR = 10.46, *x*^2^ = 17.96).

It was demonstrated in this sub-group analysis that no particular trends or patterns were observed across therapeutic class. This could be possibly due to treatments having different mechanism of actions, even within the same therapeutic class.

## 4. Discussion

Among the 56 shortlisted drugs within the UK Yellow Card System, there were 6133 cardiovascular adverse drug reactions (4.78%) reported over a total of 128,214 adverse events reports. According to the criteria for signal detection defined by ROR and PRR, signals were detected for 17 drugs using ROR and for 2 drugs using PRR. This discrepancy may arise because PRR, which is based on the proportion of reports of specific adverse events among all reported events for a drug, might be biased by including other adverse events in its denominator and does not account for the underlying population at risk, whereas ROR excludes related adverse events from the control group [13].

In our study, cardiovascular adverse drug reactions were frequently reported in the UK Yellow Card System for drugs such as anthracyclines and platinum-based agents. This aligns with the literature published by Cardinale et al. and Ferroni et al. [14,15]. Anthracyclines, e.g., doxorubicin and epirubicin, have been associated with dose-dependent cardiotoxicity, leading to an increased risk of heart failure, myocardial infarction, and arrhythmias [14]. Platinum-based agents, e.g., cisplatin and carboplatin, can cause endothelial dysfunction and electrolyte imbalances, contributing to the development of cardiovascular events [15].

By highlighting drugs with over 10% cardiovascular AEs in Table 2, this information can be used to help inform clinicians regarding the need to monitor patients more closely when prescribing these treatments, particularly for individuals with pre-existing cardiovascular conditions. This percentage threshold can also help prioritise these drugs for further investigation. Epirubicin was one of the two drugs for which a signal for cardiovascular adverse drug reactions (PRR = 2.22, *x*^2^ = 118.91, No. of cases = 173, and ROR > 1 with 95% CI excludes null) was observed in both. Epirubicin is a chemotherapy drug belonging to the anthracycline class. The published literature suggests that late development of chronic cardiac failure may be related to dose, and thus, the upper limit of dosing (900 mg/m^2^) has been recommended for use [16,17]. Whilst our study was not able to evaluate dose–response relationship, the positive finding aligns with clinical knowledge. The mechanism of action of epirubicin involves the generation of free radicals, leading to oxidative stress and damage to cardiac cells. This damage thus results in changes to the heart muscle, which impair its function over time. Moreover, epirubicin interferes with mitochondrial function in cardiac cells, hence contributing to its cardiotoxic effects [18,19]. Alectinib was one of the two drugs that detected a signal for cardiovascular adverse events using both methods (PRR = 3.01, *x*^2^ = 32.36, No. of cases = 23, and ROR = 3.35, 95% CI = 2.15 to 5.21). The ROR value of alectinib in this study was comparable to the study by Waliany et al., which also detected a signal of alectinib related to cardiovascular events using the WHO pharmacovigilance database VigiBase [20]. Alectinib is a targeted therapy drug used majorly to treat NSCLC patients whose tumours are anaplastic lymphoma kinase (ALK)-positive. It is believed that alectinib works by blocking the activity of the ALK protein, thus inhibiting the proliferation of cancer cells and promoting their death [21]. Another study conducted by Niimura et al. using VigiBase also suggested that the risk of cardiovascular disease, specifically cardiac conductive disorders, pericarditis and heart failure, may be increased in alectinib [22]. Tyrosine Kinase Inhibitors (TKIs), such as EGFR inhibitors (e.g., erlotinib and gefitinib) and ALK inhibitors (e.g., crizotinib and alectinib), have significantly improved outcomes for NSCLC patients [23]. But EGFR inhibitors were reported to be associated with an increased risk of hypertension and QT interval prolongation, while ALK inhibitors were linked to bradycardia and QT prolongation. Vascular endothelial growth factor (VEGF) inhibitors, such as bevacizumab, were reported to be associated with hypertension and arterial thromboembolic events in the UK Yellow Card System. Given the identified signals, clinicians should consider implementing more rigorous cardiovascular monitoring for patients receiving treatments with cardiovascular signals. This includes but is not limited to regular blood pressure measurements, electrocardiograms (ECGs) and monitoring for signs of heart failure or arrhythmias, such as palpitations or tachycardia. Early detection of cardiovascular diseases can help mitigate risks and ensure prompt treatments as needed.

In this study, signal was detected in males aged 80–89 in doxorubicin (ROR = 13.28, 95% CI = 4.73–37.33; PRR = 8.37, *x*^2^ = 40.85). This aligned with findings from the existing literature. It was suggested that there was an increased risk of cardiovascular events associated with anthracyclines in adult males with cancer. However, whilst anthracyclines are known to be associated with cardiotoxicity, other risk factors include pre-existing cardiovascular disease [24] and male sex [25]. To date, there is no clear explanation of the sex differences in cardiac toxicity associated with anthracyclines.

There were no particular trends or patterns observed in sex or age difference across different immunotherapies in this study, whereas published research using a pharmacovigilance database indicated that older females could be risk factors for immunotherapy-associated myocarditis. However, these results might be skewed by several confounding factors, such as bias towards reporting only unusual or severe adverse events and the lower number of women treated for non-small cell lung cancer [26]. Other studies suggested that female patients had a higher risk of immunotherapy-related myocarditis, but these findings lacked consistent confirmation [27].

One of the limitations of this study is the under-reporting of ADRs. Many factors can contribute to this, including lack of awareness about the reporting system, uncertainty about whether the drug caused the reaction, or perceived time constraints. Under-reporting can impact the study by potentially underestimating the true incidence of ADRs, skewing the data, as well as reducing the reliability of the findings. In addition, being a spontaneous reporting system, it is susceptible to various biases. For instance, newer drugs might be over-reported due to increased attention whereas well-established drugs might be under-reported as their side effects are deemed “known”. There is also a potential for incomplete or inaccurate data since the reporting does not undergo stringent validation. This disparity in reporting can lead to an overemphasis on certain drug classes or adverse reactions, thereby distorting the overall signal reliability. Moreover, establishing a suspected causality between a drug and an adverse reaction based on the UK Yellow Card System can be complex because other factors, e.g., underlying diseases, concomitant medications and lifestyle habits, may also contribute to an observed ADR, and thus, causality cannot be inferred due to the observational design and potential confounding factors. It was also not possible to calculate incidence and prevalence rates as it provided limited information about the number of patients who consumed the drug but did not experience an ADR. Furthermore, there was no adjustment for multiplicity, which could lead to an increased risk of false positives, as well as the stratification resulting in low precision and thus very wide CI of ROR and PRR in age- and sex-stratified results. It was also noted that the public were encouraged to submit a Yellow Card report even if it was just a suspicion that the drug might have caused the ADR; hence, the ADRs included in the report might not necessarily be caused by the said drug [28]. Although PRR and ROR are useful tools for detecting ADRs, they have inherent limitations, such as potential false positives and variability in sub-group analyses, that must be considered.

Therefore, to validate and expand on the findings, follow-up studies, e.g., cohort studies (prospective or retrospective), randomised controlled trials (RCTs) and mechanistic investigations, could be carried out. Cohort studies allow patients to be followed over time, enabling the monitoring of cardiovascular events and adjusting for confounding factors, such as comorbidities and concomitant medications. This would provide more robust data on the true incidence of ADRs and their potential causal relationships with the drug. Also, RCTs with larger sample size with appropriate diversity representation could be designed to specifically examine cardiovascular safety endpoints in those treatments with cardiotoxicity signals from our study. Additionally, mechanistic investigations would allow understanding of the underlying molecular and physiological mechanisms through which cardiovascular effects might be induced.

## 5. Conclusions

The top 10 highest reported cardiovascular reactions, at Preferred Terms Level, were flushing, followed by hypertension, hypotension, cardiac failure, tachycardia, myocardial infarction, deep vein thrombosis, palpitations, cardiac arrest and atrial fibrillation. Seventeen drugs exhibited a ROR value (lower limit of the 95% CI) of ≥1, thus indicating an ADR signal. However, based on the criteria for signal detection by PRR, only alectinib and epirubicin showed cardiovascular signals. Among the analysed drugs, alectinib demonstrated the highest signal for potential cardiovascular ADRs, evidenced by both the highest ROR and PRR values. These results align with the published literature. Despite this, clinical studies have shown that alectinib significantly improves PFS and overall survival in patients. Hence, it is crucial to continue monitoring the real-world use of alectinib to ensure that the benefit–risk balance is maintained. In conclusion, while the current findings suggest association between certain NSCLC treatments and cardiovascular ADRs, they do not establish causality. Causality cannot be inferred due to the observational design and potential confounding factors. Therefore, these findings should serve as a basis for further research to better understand the cardiovascular safety profile of the NSCLC drugs.

## Figures and Tables

**Table 1:** A list of lung cancer (ICD-10: C34) drugs available within the Yellow Card database.

Lung Cancer (ICD-10: C34) Drugs *	Anatomical Therapeutic Chemical (ATC) Classification **
Afatinib	L01EB03
Alectinib	L01ED03
Amifostine	V03AF05
Atezolizumab	L01FF05
Avelumab	L01FF04
Bevacizumab	L01FG01
Brigatinib	L01ED04
Carboplatin	L01XA02
Cabozantinib	L01EX07
Celecoxib	L01XX33
Ceritinib	L01ED02
Cetuximab	L01FE01
Cisplatin	L01XA01
Crizotinib	L01ED01
Cyclophosphamide	L01AA01
Dabrafenib	L01EC02
Dacomitinib	L01EB07
Dasatinib	L01EA02
Docetaxel	L01CD02
Doxorubicin	L01DB01
Durvalumab	L01FF03
Entrectinib	L01EX14
Erlotinib	L01EB02
Epirubicin	L01DB03
Etoposide	L01CB01
Everolimus	L01EG02/L04AA18
Gefitinib	L01EB01
Gemcitabine	L01BC05
Ifosfamide	L01AA06
Ipilimumab	L01FX04
Irinotecan	L01CE02
Larotrectinib	L01EX12
Lorlatinib	L01ED05
Lurbinectedin	L01XX69
Methotrexate	L01BA01/L04AX03
Nintedanib	L01EX09
Nivolumab	L01FF01
Osimertinib	L01EB04
Paclitaxel	L01CD01
Panitumumab	L01FE02
Pembrolizumab	L01FF02
Pemetrexed	L01BA04
Pralsetinib	L01EX23
Ramucirumab	L01FG02
Selpercatinib	L01EX22
Sorafenib	L01EX02
Sotorasib	L01XX73
Sunitinib	L01EX01
Tepotinib	L01EX21
Topotecan	L01CE01
Trametinib	L01EE01
Trastuzumab	L01FD01
Veliparib	L01XK05
Vemurafenib	L01EC01
Vincristine	L01CA02
Vinorelbine	L01CA04

* ICD-10: International Classification of Diseases 10th Revision. ** For more detailed information on ATC Classification, refer to https://www.who.int/tools/atc-ddd-toolkit/atc-classification (accessed on 27 April 2022).

**Table 2:** An overview of the total number of suspected adverse drug reactions reported for the 56 drugs and their corresponding number of cardiovascular events.

Drug	Total Number of Adverse Event Reports	Total Number of Cardiovascular (CV) Adverse Event Reports (%)	
Alectinib	160	23	14.38%
Epirubicin	1652	173	10.47%
Doxorubicin	4408	376	8.53%
Avelumab	843	69	8.19%
Selpercatinib	172	14	8.14%
Dasatinib	1289	95	7.37%
Celecoxib	5039	335	6.65%
Pemetrexed	1359	90	6.62%
Gemcitabine	2430	160	6.58%
Nintedanib	995	65	6.53%
Paclitaxel	5211	336	6.45%
Cisplatin	3611	225	6.23%
Docetaxel	7692	475	6.18%
Carboplatin	5235	318	6.07%
Vinorelbine	1920	114	5.94%
Sunitinib	2555	147	5.75%
Bevacizumab	3945	210	5.32%
Trastuzumab	13,863	718	5.18%
Crizotinib	851	43	5.05%
Cyclophosphamide	8394	408	4.86%
Osimertinib	437	21	4.81%
Gefitinib	439	21	4.78%
Vincristine	3835	181	4.72%
Cetuximab	1061	50	4.71%
Etoposide	3603	165	4.58%
Sorafenib	820	37	4.51%
Ceritinib	180	8	4.44%
Irinotecan	1729	76	4.40%
Vemurafenib	569	25	4.39%
Pembrolizumab	3292	143	4.34%
Dacomitinib	48	2	4.17%
Nivolumab	3199	132	4.13%
Brigatinib	130	5	3.85%
Ipilimumab	2305	86	3.73%
Erlotinib	1155	43	3.72%
Ifosfamide	1491	54	3.62%
Entrectinib	56	2	3.57%
Durvalumab	286	10	3.50%
Lorlatinib	183	5	2.73%
Dabrafenib	1767	48	2.72%
Cabozantinib	1560	41	2.63%
Tepotinib	77	2	2.60%
Topotecan	670	17	2.54%
Trametinib	1581	37	2.34%
Methotrexate	18,709	418	2.23%
Larotrectinib	46	1	2.17%
Afatinib	434	9	2.07%
Everolimus	3949	67	1.70%
Panitumumab	617	9	1.46%
Atezolizumab	2133	24	1.13%
Amifostine	3	0	0.00%
Lurbinectedin	2	0	0.00%
Pralsetinib	33	0	0.00%
Ramucirumab	40	0	0.00%
Sotorasib	130	0	0.00%
Veliparib	21	0	0.00%
Total	128,214	6133	4.78%

## Data Availability

The data presented in this study are available on request from the corresponding author.

## Supplementary Materials

The following supporting information can be downloaded at: https://www.mdpi.com/article/10.3390/cancers16244223/s1, Table S1: List of variables extracted from the Yellow Card database (adapted from MHRA). Table S2: Patients’ demographics and seriousness of each cardiovascular adverse event reported by drug.

## References

[1] Ferlay J., Ervik M., Lam F., Laversanne M., Colombet M., Mery L., Piñeros M., Znaor A., Soerjomataram I., Bray F. (2024). Global Cancer Observatory: Cancer Today.

[2] IQVIA (2023). Global Oncology Trends 2023.

[3] IQVIA (2021). Global Oncology Trends 2021.

[4] Tan C., Tasaka H., Yu K.-P., Murphy M.L., Karnofsky D.A. (1967). Daunomycin, an antitumor antibiotic, in the treatment of neoplastic disease.Clinical evaluation with special reference to childhood leukemia. Cancer.

[5] Kerkelä R., Grazette L., Yacobi R., Iliescu C., Patten R., Beahm C., Walters B., Shevtsov S., Pesant S., Clubb F.J. (2006). Cardiotoxicity of the cancer therapeutic agent imatinib mesylate. Nat. Med..

[6] Santoni M., Guerra F., Conti A., Lucarelli A., Rinaldi S., Belvederesi L., Capucci A., Berardi R. (2017). Incidence and risk of cardiotoxicity in cancer patients treated with targeted therapies. Cancer Treat. Rev..

[7] Härmark L., van Grootheest A.C. (2008). Pharmacovigilance: Methods, recent developments and future perspectives. Eur. J. Clin. Pharmacol..

[8] Schad F., Thronicke A. (2022). Real-World Evidence—Current Developments and Perspectives. Int. J. Environ. Res. Public Health.

[9] Shuttleworth P., Baker J., Clark E. (2023). Under-reporting of gastrointestinal bleeding associated with anticoagulant use using the UK Yellow Card Scheme. Int. J. Clin. Pharm..

[10] Nour S., Plourde G. (2019). Pharmacovigilance. Pharmacoepidemiology and Pharmacovigilance.

[11] Evans S.J.W., Waller P.C., Davis S. (2001). Use of proportional reporting ratios (PRRs) for signal generation from spontaneous adverse drug reaction reports. Pharmacoepidemiol. Drug Saf..

[12] Anand K., Ensor J., Trachtenberg B., Bernicker E.H. (2019). Osimertinib-Induced Cardiotoxicity. JACC Cardiooncol..

[13] Rothman K.J., Lanes S., Sacks S.T. (2004). The reporting odds ratio and its advantages over the proportional reporting ratio. Pharmacoepidemiol. Drug Saf..

[14] Cardinale D., Iacopo F., Cipolla C.M. (2020). Cardiotoxicity of Anthracyclines. Front. Cardiovasc. Med..

[15] Ferroni P., Della-Morte D., Palmirotta R., McClendon M., Testa G., Abete P., Rengo F., Rundek T., Guadagni F., Roselli M. (2011). Platinum-based compounds and risk for cardiovascular toxicity in the elderly: Role of the antioxidants in chemoprevention. Rejuvenation Res..

[16] Appel J.M., Nielsen D., Zerahn B., Jensen B.V., Skagen K. (2007). Anthracycline-induced chronic cardiotoxicity and heart failure. Acta Oncol..

[17] Ryberg M., Nielsen D., Skovsgaard T., Hansen J., Jensen B.V., Dombernowsky P. (1998). Epirubicin cardiotoxicity: An analysis of 469 patients with metastatic breast cancer. J. Clin. Oncol..

[18] Cersosimo R.J., Hong W.K. (1986). Epirubicin: A review of the pharmacology, clinical activity, and adverse effects of an adriamycin analogue. J. Clin. Oncol..

[19] Plosker G.L., Faulds D. (1993). Epirubicin. A review of its pharmacodynamic and pharmacokinetic properties, and therapeutic use in cancer chemotherapy. Drugs.

[20] Waliany S., Zhu H., Wakelee H., Padda S.K., Das M., Ramchandran K., Myall N.J., Chen T., Witteles R.M., Neal J.W. (2021). Pharmacovigilance Analysis of Cardiac Toxicities Associated with Targeted Therapies for Metastatic NSCLC. J. Thorac. Oncol..

[21] Paik J., Dhillon S. (2018). Alectinib: A Review in Advanced, ALK-Positive NSCLC. Drugs.

[22] Niimura T., Miyata K., Hamano H., Nounin Y., Unten H., Yoshino M., Mitsuboshi S., Aizawa F., Yagi K., Koyama T. (2023). Cardiovascular Toxicities Associated with Anaplastic Lymphoma Kinase Inhibitors: A Disproportionality Analysis of the WHO Pharmacovigilance Database (VigiBase). Drug Saf..

[23] Chang W.-T., Lin H.-W., Chang T.-C., Lin S.-H., Li Y.-H. (2023). Assessment of Tyrosine Kinase Inhibitors and Survival and Cardiovascular Outcomes of Patients with Non–Small Cell Lung Cancer in Taiwan. JAMA Netw. Open.

[24] Wang L., Tan T.C., Halpern E.F., Neilan T.G., Francis S.A., Picard M.H., Fei H., Hochberg E.P., Abramson J.S., Weyman A.E. (2015). Major Cardiac Events and the Value of Echocardiographic Evaluation in Patients Receiving Anthracycline-Based Chemotherapy. Am. J. Cardiol..

[25] Myrehaug S., Pintilie M., Yun L., Crump M., Tsang R.W., Meyer R.M., Sussman J., Yu E., Hodgson D.C. (2010). A population-based study of cardiac morbidity among Hodgkin lymphoma patients with preexisting heart disease. Blood.

[26] Zamami Y., Niimura T., Okada N., Koyama T., Fukushima K., Izawa-Ishizawa Y., Ishizawa K. (2019). Factors Associated with Immune Checkpoint Inhibitor-Related Myocarditis. JAMA Oncol..

[27] Wilcox N.S., Rotz S.J., Mullen M., Song E.J., Hamilton B.K., Moslehi J., Armenian S.H., Wu J.C., Rhee J.-W., Ky B. (2022). Sex-Specific Cardiovascular Risks of Cancer and Its Therapies. Circ. Res..

[28] Alomar M., Tawfiq A.M., Hassan N., Palaian S. (2020). Post marketing surveillance of suspected adverse drug reactions through spontaneous reporting: Current status, challenges and the future. Ther. Adv. Drug Saf..

