# Supplementary material for: Cancer-Therapy-Induced Cardiotoxicity: Results of the Analysis of the UK Yellow Card Adverse Drug Reaction (ADR) Reporting
Source: Cancers (Basel). 2024 Dec 18;16(24):4223. doi: 10.3390/cancers16244223 (PMC11674627; doi:10.3390/cancers16244223)
Supplement: Supplementary file 1 [file cancers-16-04223-s001.zip › cancers-3336343-supplementary.pdf]

Table S1: List of variables extracted from the Yellow Card database (adapted from MHRA).

Column Name	Column Description	Extracted from the Following Source Table
ADR	Sequential number identifying a single ADR report	CASE
SEX	Patient sex: "Male", "Female", "Unknown"	CASE
AGE_10	Patient age at the time of the ADR. This is expressed in 10-year bands with the lowest level of the band displayed in the file e.g.,<br>0 represents ages less than 10 years (0-9 years)<br>10 represents ages of 10 years or higher, but less than 20 years (10-19 years)<br>20 represents ages of 20 years or higher, but less than 30 years (20-29 years)	CASE
RECVD_YEAR	Year in which the ADR was first received by the MHRA	CASE
SENDER_TYPE	Describes the source of the ADR reports submitted to the MHRA:<br>"Indirect" if the ADR was reported to the MHRA by a pharmaceutical company<br>"Direct" if the ADR was reported to the MHRA by a healthcare professional or member of the public	CASE
CONSUMER_YN	Indicates if the ADR was reported by the consumer (the patient, their parent or a carer) of the product<br>"Y" if the ADR was reported by a consumer or a lawyer.<br>"N" if the ADR was not reported by a consumer or a lawyer	CASE
HCP_YN	Indicates if the ADR was reported by a healthcare professional. (examples of healthcare professionals include: Hospital Doctor, GP, pharmacist, nurse, etc)<br>"Y" if the ADR was reported by a health professional<br>"N" if the ADR was reported by a non-health professional<br><br>Please note HCP_YN and Consumer_YN are not exclusive categories; an ADR report can be sent to the MHRA with details of both a consumer and healthcare professional reporter	CASE
NONSERIOUS_SERIOUS_FATAL_NSF	Indicates the seriousness of the ADR report<br>"F" if the ADR was fatal<br>"S" if the ADR was serious but not fatal.<br>"N" if the ADR was non-serious.	CASE
ADR	Sequential number identifying a single ADR report	DRUG
SEQ	Sequential number identifying each reported administration of the suspect drug in question	DRUG
ROUTE	The route of administration for the drug (e.g. oral, intravenous, etc).<br>Drug routes that appear in fewer than 5 ADR reports (or 1% of ADR reports for drugs with more than 500 ADRs) are displayed as "Routes with few reports"	DRUG
MULTICONST	Describes whether the drug in each ADR report contains more than one active ingredient<br>"Y" if the drug substance of interest in the IDAP is one active ingredient in a multi- constituent product.<br>"N" if the drug substance of interest in the IDAP is the only active ingredient in the product.	DRUG
NONSERIOUS_SERIOUS_FATAL_NSF	Indicates the seriousness of the ADR report. This field is also in CASE.csv<br>"F" if the ADR was fatal<br>"S" if the ADR was serious but not fatal.<br>"N" if the ADR was non-serious.	DRUG

ADR	Sequential number identifying a single ADR report	EVENT
SEQ	Sequential number identifying each adverse reaction within the ADR report	EVENT
PT	MedDRA Preferred Term, describing the adverse reaction	EVENT
HLT	MedDRA High Level Term which contains the corresponding Preferred Term	EVENT
HLGT	MedDRA High Level Group Term which contains the corresponding High Level Term	EVENT
SOC_ABBREV	MedDRA System Organ Class which contains the corresponding High Level Group Term	EVENT
FATAL_YN	Describes whether the adverse reaction had a fatal outcome<br>"Y" if the reaction was fatal<br>"N" if the reaction was not fatal	EVENT
DRUG_NAME	The name of the drug substance of interest in this IDAP report	INFO
DATA_EXTRACT_VERSION	The software version used to generate the data in the report	INFO
DATA_EXTRACTION_DATE	The date when the data for this report were produced. Please note that a drug with less frequent ADRs reported may have an older date because new IDAP datasets are only created after new ADR reports for the drug have been received.	INFO
MEDDRA_VERSION	The version of the MedDRA dictionary that was active on the data extraction date. Values are 2-3 digit numeric strings e.g. "19" (for MedDRA version 19.0), "181" (for MedDRA version 18.1) etc.	INFO
REPORT_TYPE	The level within the dictionary hierarchy of the information included on the IDAP. Not all levels are currently routinely published online. If you require further information, please contact the MHRA. Levels include:<br>"Combined substance" - A drug that contains one or more active ingredients<br>"Substance" - An active ingredient, regardless of whether it is administered by itself or in combination with other ingredients,<br>"Substance Variant" - A drug chemical variant of a substance<br>"PBG" - Product Brand Generic, a family name for a group of related products	INFO
COUNTRY	The country where the ADRs in the IDAP were reported.	INFO
MULTI_CONSTITUENT_YN	"N" indicates that the row is summarising single-constituent products<br>"Y" indicates that the row is summarising multi-constituent products	SUMMARY
PBG	A list of the single-constituent or multi-constituent products that are summarised in the report. The products are identified by their "Product Brand Generic" (PBG) names.	SUMMARY

Table S2. Patients' demographics and seriousness of each cardiovascular adverse event reported by drug.

Drug	Total number of CV reports	Gender						Age																				Seriousness					
		Female		Male		Unknown		0		10		20		30		40		50		60		70		80		90		Fatal		Non-Serious		Serious	
**Afatinib**	9	3	33.33%	6	66.67%	0	0.00%	0	0.00%	0	0.00%	0	0.00%	0	0.00%	1	11.11%	2	22.22%	1	11.11%	0	0.00%	4	44.44%	1	11.11%	0	0.00%	0	0.00%	9	100.00%
**Alectinib**	23	7	30.43%	14	60.87%	2	8.70%	0	0.00%	0	0.00%	0	0.00%	1	4.35%	2	8.70%	10	43.48%	3	13.04%	0	0.00%	0	0.00%	0	0.00%	0	0.00%	0	0.00%	23	100.00%
**Atezolizumab**	24	9	37.50%	14	58.33%	1	4.17%	0	0.00%	0	0.00%	0	0.00%	0	0.00%	1	4.17%	7	29.17%	3	12.50%	10	41.67%	0	0.00%	0	0.00%	3	12.50%	1	4.17%	20	83.33%
**Avelumab**	69	13	18.84%	55	79.71%	1	1.45%	0	0.00%	0	0.00%	0	0.00%	0	0.00%	3	4.35%	21	30.43%	24	34.78%	8	11.59%	10	14.49%	0	0.00%	3	4.35%	1	1.45%	65	94.20%
**Bevacizumab**	210	108	51.43%	91	43.33%	11	5.24%	2	0.95%	3	1.43%	0	0.00%	5	2.38%	15	7.14%	35	16.67%	63	30.00%	21	10.00%	8	3.81%	0	0.00%	25	11.90%	1	0.48%	184	87.62%
**Brigatinib**	5	2	40.00%	1	20.00%	2	40.00%	0	0.00%	0	0.00%	0	0.00%	0	0.00%	0	0.00%	1	20.00%	0	0.00%	0	0.00%	0	0.00%	0	0.00%	0	0.00%	0	0.00%	5	100.00%
**Cabozantinib**	41	15	36.59%	21	51.22%	5	12.20%	0	0.00%	0	0.00%	0	0.00%	1	2.44%	6	14.63%	9	21.95%	19	46.34%	4	9.76%	1	2.44%	0	0.00%	6	14.63%	1	2.44%	34	82.93%
**Carboplatin**	318	242	76.10%	59	18.55%	17	5.35%	7	2.20%	0	0.00%	5	1.57%	12	3.77%	35	11.01%	60	18.87%	81	25.47%	52	16.35%	10	3.14%	0	0.00%	38	11.95%	34	10.69%	246	77.36%
**Celecoxib**	335	193	57.61%	131	39.10%	11	3.28%	0	0.00%	0	0.00%	1	0.30%	12	3.58%	28	8.36%	65	19.40%	85	25.37%	60	17.91%	36	10.75%	9	2.69%	32	9.55%	52	15.52%	251	74.93%
**Ceritinib**	8	8	100.00%	0	0.00%	0	0.00%	0	0.00%	0	0.00%	0	0.00%	1	12.50%	0	0.00%	5	62.50%	1	12.50%	0	0.00%	0	0.00%	0	0.00%	1	12.50%	0	0.00%	7	87.50%
**Cetuximab**	50	14	28.00%	35	70.00%	1	2.00%	0	0.00%	0	0.00%	0	0.00%	4	8.00%	1	2.00%	10	20.00%	13	26.00%	7	14.00%	0	0.00%	0	0.00%	10	20.00%	5	10.00%	35	70.00%
**Cisplatin**	225	83	36.89%	134	59.56%	8	3.56%	7	3.11%	0	0.00%	7	3.11%	23	10.22%	35	15.56%	48	21.33%	63	28.00%	12	5.33%	0	0.00%	0	0.00%	33	14.67%	4	1.78%	188	83.56%

**Crizotinib**	43	28	65.12%	15	34.88%	0	0.00%	0	0.00%	0	0.00%	4	9.30%	2	4.65%	11	25.58%	3	6.98%	5	11.63%	10	23.26%	1	2.33%	0	0.00%	8	18.60%	1	2.33%	34	79.07%
**Cyclophosphamide**	408	208	50.98%	148	36.27%	52	12.75%	34	8.33%	15	3.68%	9	2.21%	31	7.60%	72	17.65%	69	16.91%	39	9.56%	37	9.07%	9	2.21%	0	0.00%	71	17.40%	4	0.98%	333	81.62%
**Dabrafenib**	48	9	18.75%	34	70.83%	5	10.42%	0	0.00%	0	0.00%	2	4.17%	2	4.17%	0	0.00%	2	4.17%	14	29.17%	9	18.75%	1	2.08%	0	0.00%	11	22.92%	0	0.00%	37	77.08%
**Dacomitinib**	2	2	100.00%	0	0.00%	0	0.00%	0	0.00%	0	0.00%	0	0.00%	1	50.00%	0	0.00%	0	0.00%	0	0.00%	1	50.00%	0	0.00%	0	0.00%	1	50.00%	0	0.00%	1	50.00%
**Dasatinib**	95	40	42.11%	50	52.63%	5	5.26%	0	0.00%	0	0.00%	1	1.05%	6	6.32%	9	9.47%	11	11.58%	28	29.47%	14	14.74%	2	2.11%	0	0.00%	7	7.37%	1	1.05%	87	91.58%
**Docetaxel**	475	346	72.84%	109	22.95%	20	4.21%	0	0.00%	0	0.00%	1	0.21%	38	8.00%	68	14.32%	118	24.84%	85	17.89%	51	10.74%	7	1.47%	0	0.00%	87	18.32%	40	8.42%	348	73.26%
**Doxorubicin**	376	191	50.80%	140	37.23%	45	11.97%	23	6.12%	28	7.45%	11	2.93%	14	3.72%	47	12.50%	72	19.15%	42	11.17%	44	11.70%	9	2.39%	0	0.00%	82	21.81%	10	2.66%	284	75.53%
**Durvalumab**	10	4	40.00%	4	40.00%	2	20.00%	0	0.00%	0	0.00%	0	0.00%	0	0.00%	0	0.00%	1	10.00%	2	20.00%	2	20.00%	0	0.00%	0	0.00%	4	40.00%	0	0.00%	6	60.00%
**Entrectinib**	2	2	100.00%	0	0.00%	0	0.00%	0	0.00%	0	0.00%	0	0.00%	0	0.00%	0	0.00%	2	100.00%	0	0.00%	0	0.00%	0	0.00%	0	0.00%	0	0.00%	0	0.00%	2	100.00%
**Epirubicin**	173	137	79.19%	30	17.34%	6	3.47%	2	1.16%	7	4.05%	6	3.47%	11	6.36%	29	16.76%	43	24.86%	14	8.09%	11	6.36%	0	0.00%	0	0.00%	18	10.40%	7	4.05%	148	85.55%
**Erlotinib**	43	22	51.16%	19	44.19%	2	4.65%	0	0.00%	0	0.00%	0	0.00%	0	0.00%	2	4.65%	8	18.60%	13	30.23%	10	23.26%	1	2.33%	0	0.00%	14	32.56%	0	0.00%	29	67.44%
**Etoposide**	165	81	49.09%	76	46.06%	8	4.85%	29	17.58%	5	3.03%	8	4.85%	20	12.12%	14	8.48%	15	9.09%	23	13.94%	23	13.94%	0	0.00%	0	0.00%	20	12.12%	6	3.64%	139	84.24%
**Everolimus**	67	55	82.09%	7	10.45%	5	7.46%	17	25.37%	0	0.00%	0	0.00%	2	2.99%	3	4.48%	15	22.39%	10	14.93%	5	7.46%	1	1.49%	0	0.00%	13	19.40%	0	0.00%	54	80.60%
**Gefitinib**	21	13	61.90%	8	38.10%	0	0.00%	0	0.00%	0	0.00%	0	0.00%	0	0.00%	0	0.00%	2	9.52%	5	23.81%	7	33.33%	5	23.81%	0	0.00%	3	14.29%	0	0.00%	18	85.71%
**Gemcitabine**	160	73	45.63%	67	41.88%	20	12.50%	0	0.00%	2	1.25%	1	0.63%	1	0.63%	9	5.63%	25	15.63%	60	37.50%	26	16.25%	5	3.13%	0	0.00%	30	18.75%	11	6.88%	119	74.38%

**Ifosfamide**	54	31	57.41%	21	38.89%	2	3.70%	3	5.56%	13	24.07%	1	1.85%	6	11.11%	4	7.41%	8	14.81%	7	12.96%	0	0.00%	0	0.00%	0	0.00%	6	11.11%	2	3.70%	46	85.19%
**Ipilimumab**	86	30	34.88%	51	59.30%	5	5.81%	0	0.00%	0	0.00%	0	0.00%	4	4.65%	0	0.00%	8	9.30%	39	45.35%	19	22.09%	2	2.33%	0	0.00%	29	33.72%	0	0.00%	57	66.28%
**Irinotecan**	76	31	40.79%	44	57.89%	1	1.32%	0	0.00%	0	0.00%	0	0.00%	3	3.95%	5	6.58%	27	35.53%	18	23.68%	8	10.53%	0	0.00%	0	0.00%	14	18.42%	5	6.58%	57	75.00%
**Larotrectinib**	1	1	100.00%	0	0.00%	0	0.00%	0	0.00%	0	0.00%	0	0.00%	0	0.00%	0	0.00%	1	100.00%	0	0.00%	0	0.00%	0	0.00%	0	0.00%	0	0.00%	0	0.00%	1	100.00%
**Lorlatinib**	5	0	0.00%	5	100.00%	0	0.00%	0	0.00%	0	0.00%	0	0.00%	0	0.00%	0	0.00%	1	20.00%	2	40.00%	0	0.00%	0	0.00%	0	0.00%	0	0.00%	0	0.00%	5	100.00%
**Methotrexate**	418	229	54.78%	169	40.43%	20	4.78%	24	5.74%	40	9.57%	19	4.55%	21	5.02%	39	9.33%	58	13.88%	82	19.62%	59	14.11%	14	3.35%	1	0.24%	96	22.97%	11	2.63%	311	74.40%
**Nintedanib**	65	8	12.31%	52	80.00%	5	7.69%	0	0.00%	0	0.00%	0	0.00%	0	0.00%	0	0.00%	4	6.15%	17	26.15%	27	41.54%	9	13.85%	0	0.00%	7	10.77%	1	1.54%	57	87.69%
**Nivolumab**	132	45	34.09%	83	62.88%	4	3.03%	0	0.00%	0	0.00%	4	3.03%	1	0.76%	7	5.30%	9	6.82%	50	37.88%	25	18.94%	5	3.79%	0	0.00%	29	21.97%	5	3.79%	98	74.24%
**Osimertinib**	21	14	66.67%	7	33.33%	0	0.00%	0	0.00%	0	0.00%	0	0.00%	0	0.00%	0	0.00%	1	4.76%	10	47.62%	6	28.57%	2	9.52%	0	0.00%	10	47.62%	0	0.00%	11	52.38%
**Paclitaxel**	336	283	84.23%	33	9.82%	20	5.95%	0	0.00%	1	0.30%	0	0.00%	23	6.85%	44	13.10%	65	19.35%	92	27.38%	51	15.18%	5	1.49%	0	0.00%	39	11.61%	53	15.77%	244	72.62%
**Panitumumab**	9	6	66.67%	3	33.33%	0	0.00%	0	0.00%	0	0.00%	0	0.00%	0	0.00%	1	11.11%	5	55.56%	0	0.00%	0	0.00%	0	0.00%	0	0.00%	1	11.11%	0	0.00%	8	88.89%
**Pembrolizumab**	143	62	43.36%	79	55.24%	2	1.40%	0	0.00%	0	0.00%	0	0.00%	2	1.40%	4	2.80%	21	14.69%	21	14.69%	60	41.96%	15	10.49%	2	1.40%	42	29.37%	2	1.40%	99	69.23%
**Pemetrexed**	90	41	45.56%	48	53.33%	1	1.11%	0	0.00%	0	0.00%	0	0.00%	2	2.22%	7	7.78%	15	16.67%	42	46.67%	16	17.78%	0	0.00%	0	0.00%	21	23.33%	0	0.00%	69	76.67%
**Selpercatinib**	14	7	50.00%	7	50.00%	0	0.00%	0	0.00%	0	0.00%	0	0.00%	0	0.00%	0	0.00%	4	28.57%	4	28.57%	2	14.29%	0	0.00%	0	0.00%	0	0.00%	0	0.00%	14	100.00%
**Sorafenib**	37	13	35.14%	21	56.76%	3	8.11%	6	16.22%	0	0.00%	0	0.00%	0	0.00%	0	0.00%	1	2.70%	6	16.22%	8	21.62%	0	0.00%	0	0.00%	4	10.81%	0	0.00%	33	89.19%

**Sunitinib**	147	50	34.01%	95	64.63%	2	1.36%	0	0.00%	0	0.00%	0	0.00%	4	2.72%	12	8.16%	24	16.33%	42	28.57%	36	24.49%	14	9.52%	1	0.68%	26	17.69%	1	0.68%	120	81.63%
**Tepotinib**	2	2	100.00%	0	0.00%	0	0.00%	0	0.00%	0	0.00%	0	0.00%	0	0.00%	0	0.00%	0	0.00%	0	0.00%	1	50.00%	0	0.00%	0	0.00%	0	0.00%	1	50.00%	1	50.00%
**Topotecan**	17	15	88.24%	2	11.76%	0	0.00%	6	35.29%	0	0.00%	0	0.00%	0	0.00%	0	0.00%	3	17.65%	4	23.53%	0	0.00%	0	0.00%	0	0.00%	2	11.76%	0	0.00%	15	88.24%
**Trametinib**	37	8	21.62%	25	67.57%	4	10.81%	2	5.41%	0	0.00%	2	5.41%	2	5.41%	0	0.00%	2	5.41%	10	27.03%	5	13.51%	1	2.70%	0	0.00%	7	18.92%	0	0.00%	30	81.08%
**Trastuzumab**	718	692	96.38%	4	0.56%	22	3.06%	0	0.00%	0	0.00%	2	0.28%	76	10.58%	127	17.69%	140	19.50%	99	13.79%	51	7.10%	8	1.11%	0	0.00%	120	16.71%	11	1.53%	587	81.75%
**Vemurafenib**	25	12	48.00%	4	16.00%	9	36.00%	0	0.00%	0	0.00%	1	4.00%	0	0.00%	1	4.00%	0	0.00%	12	48.00%	8	32.00%	2	8.00%	0	0.00%	5	20.00%	0	0.00%	20	80.00%
**Vincristine**	181	51	28.18%	107	59.12%	23	12.71%	10	5.52%	18	9.94%	3	1.66%	9	4.97%	20	11.05%	32	17.68%	17	9.39%	22	12.15%	8	4.42%	0	0.00%	35	19.34%	1	0.55%	145	80.11%
**Vinorelbine**	114	72	63.16%	42	36.84%	0	0.00%	0	0.00%	1	0.88%	0	0.00%	2	1.75%	11	9.65%	41	35.96%	16	14.04%	17	14.91%	3	2.63%	0	0.00%	26	22.81%	1	0.88%	87	76.32%
